# 2148. No Device, But Still a Problem: A Five Year Review of Healthcare Associated Bloodstream and Urinary Tract Infections in a Children’s Hospital

**DOI:** 10.1093/ofid/ofac492.1768

**Published:** 2022-12-15

**Authors:** Amanda Schafenacker, Wendi Gornick, Beth Huff, Jasjit Singh

**Affiliations:** UC Irvine / Children's Hospital Orange County, Orange, California; Children's Hospital Orange County, Orange, California; Children's Hospital Orange County, Orange, California; CHOC Children's Hospital, Orange, California

## Abstract

**Background:**

Central line-associated bloodstream infection (CLABSI) and catheter-associated urinary tract infection (CAUTI) continue to be the focus of external reporting of healthcare associated infections based on CDC-NHSN criteria. As facilities decrease CLABSI and CAUTI, we sought to understand our non-central line bloodstream infections (NCLABSI) and non-catheter urinary tract infections (NCAUTI).

**Methods:**

We retrospectively reviewed total healthcare associated bloodstream infections (HABSI) (NCLABSI + CLABSI) and healthcare associated urinary tract infections (HAUTI) (NCAUTI + CAUTI) from July 1, 2016 to June 30, 2021 at our 334-bed quaternary care Children’s Hospital. CLABSI and CAUTI were both defined using CDC-NHSN criteria. Epidemiologic and microbiologic data, total antibiotic days related to infection, and mortality were analyzed for each subgroup.

**Results:**

In a 5-year period, 255 patients were identified with HABSI and HAUTI; 164 were HABSI (26% NCLABSI, 74% CLABSI) and 91 were HAUTI (79% NCAUTI, 21% CAUTI). While our NCLABSI, CLABSI, and CAUTI infections per fiscal year (FY) have remained relatively stable, our NCAUTIs have increased since FY17 (Figure 1). Total antibiotic days for NCLABSI versus CLABSI and for NCAUTI versus CAUTI were similar (Figure 2). *Staphylococcus aureus* was the predominant pathogen in all HABSIs (16% CLABSI, 31% NCLABSI). *Pseudomonas aeruginosa* was seen in 15% of CLABSIs but in no NCLABSIs. *P. aeruginosa* was seen in both CAUTI (32%) and NCAUTI (11%). *Escherichia coli* was the predominant pathogen in NCAUTI (39%) (Figure 3). There were 2 NCLABSI (5%), 16 CLABSI (13%), 2 NCAUTI (3%) and 2 CAUTI (11%) patient deaths during hospitalization.

**Figure:**
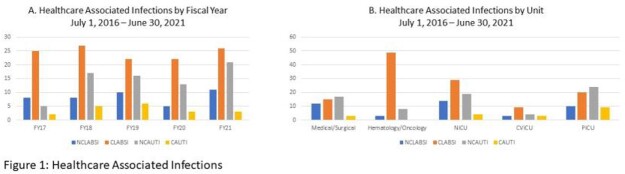

**Figure:**
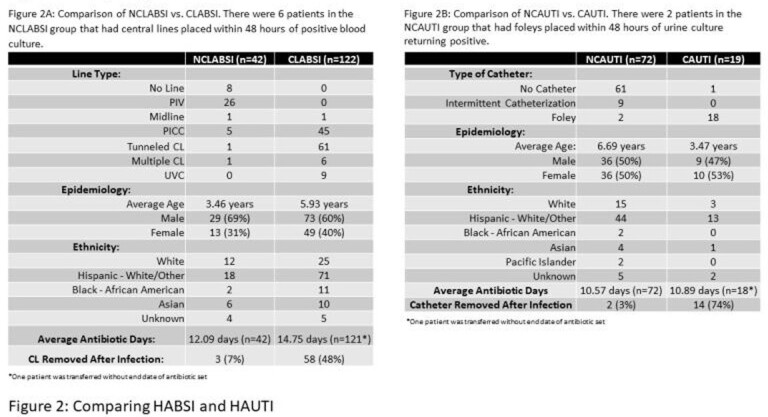

**Figure:**
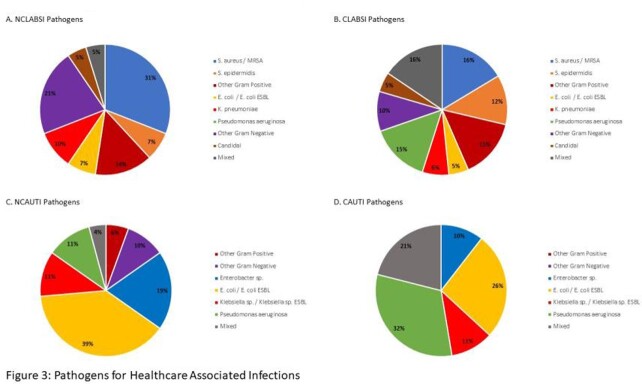

**Conclusion:**

Our data shows that non-device related healthcare associated infections are a significant proportion of our total healthcare associated infections, comprising one quarter of HABSIs and three quarters of HAUTIs. In addition, *P. aeruginosa*, typically an important pathogen in device-associated infections, also contributes to non-device associated infections. We feel that total HABSI and HAUTI may provide more objective measures to report. Infection prevention measures to decrease NCLABSI and NCAUTI also need attention.

**Disclosures:**

**All Authors**: No reported disclosures.

